# Auditory perception bias in speech imitation

**DOI:** 10.3389/fpsyg.2013.00826

**Published:** 2013-11-05

**Authors:** Marie Postma-Nilsenová, Eric Postma

**Affiliations:** Department of Communication and Information Sciences, Tilburg Center for Cognition and Communication, Tilburg UniversityTilburg, Netherlands

**Keywords:** pitch, fundamental frequency, imitation, Heschl's gyrus, missing fundamental

## Abstract

In an experimental study, we explored the role of auditory perception bias in vocal pitch imitation. Psychoacoustic tasks involving a missing fundamental indicate that some listeners are attuned to the relationship between all the higher harmonics present in the signal, which supports their perception of the fundamental frequency (the primary acoustic correlate of pitch). Other listeners focus on the lowest harmonic constituents of the complex sound signal which may hamper the perception of the fundamental. These two listener types are referred to as *fundamental* and *spectral* listeners, respectively. We hypothesized that the individual differences in speakers' capacity to imitate *F*_0_ found in earlier studies, may at least partly be due to the capacity to extract information about *F*_0_ from the speech signal. Participants' auditory perception bias was determined with a standard missing fundamental perceptual test. Subsequently, speech data were collected in a shadowing task with two conditions, one with a full speech signal and one with high-pass filtered speech above 300 Hz. The results showed that perception bias toward fundamental frequency was related to the degree of *F*_0_ imitation. The effect was stronger in the condition with high-pass filtered speech. The experimental outcomes suggest advantages for fundamental listeners in communicative situations where *F*_0_ imitation is used as a behavioral cue. Future research needs to determine to what extent auditory perception bias may be related to other individual properties known to improve imitation, such as phonetic talent.

## Introduction

Due to a plethora of linguistic and social functions, vocal pitch imitation plays a central role in human interaction. In language use, pitch, the perceptual correlate of fundamental frequency (*F*_0_) typically located between 50–500 Hz in human speech signal, encodes linguistic information regarding speech act and sentence types (Nilsenová, 2007), information structure, and, in many languages, lexical meanings (Ladd, 1996). Pitch imitation arguably accelerates acquisition of these linguistic functions because it is faster than a individual, i.e., trial-and-error based, discovery (Meltzoff et al., 2009). Imitation of phonetic features has also been found to improve speech comprehension (Adank et al., 2010). Listeners who mimicked a novel pronunciation of a sentence improved their subsequent speech reception thresholds for the sentence in a condition with background noise. Next to its linguistic functions, pitch is also the most important vocal source of information regarding emotions, stands and attitudes of the speaker (Juslin and Laukka, 2003; Ververidis and Kotropoulos, 2006). The *F*_0_ region provides acoustic information for imitation exploited in promoting social convergence and status accommodation (Gregory and Hoyt, 1982; Gregory, 1983; Gregory et al., 1993; Gregory and Webster, 1996; Gregory et al., 1997; Haas and Gregory, 2005; Pardo, 2006) and expressing ingroup–outgroup bias (Babel, 2009; Pardo et al., 2012). Speakers who are perceived as attractive, likable and/or dominant influence listeners' pitch output, and pitch convergence can be seen as an indicator of cooperative behavior in communication dyads (Nilsenová and Swerts, 2012; Okada et al., 2012). Pitch divergence, on the other hand, suggests that speakers may wish to be viewed as dissimilar and increase social distance between themselves (Giles, 1973). The capacity to perceive the fundamental frequency in the speech signal correctly and to adapt one's own pitch production according to one's linguistic and social goals is thus a core communicative skill (Giles and Coupland, 1991).

The results of a range of experimental studies suggest that speakers effortlessly imitate and converge to the phonetic properties of recently heard speech (Natale, 1975; Shockley et al., 2004; Pardo, 2006; Delvaux and Soquet, 2007; Gentilucci and Bernardis, 2007; Nielsen, 2011), including pitch (Goldinger, 1998; Babel and Bulatov, 2012; Gorisch et al., 2012). However, as noted by Babel and Bulatov, (2012), in the context of the standard shadowing paradigm, large individual differences can be found in the degree of pitch imitation—with only some participants actually converging to the *F*_0_ of the model talker (Babel and Bulatov, 2012, p. 240). The proposal of our study is that individual variation in the imitation of pitch is, at least partly, due to basic acoustic perceptual mechanisms that also influence pitch production.

Most speech imitation studies assume that there exist few individual differences among healthy hearing subjects with respect to the low-level processing of speech signal. However, past psychoacoustic research involving stimuli with a missing fundamental indicated that there is a difference between two auditory perceptual extremes, sometimes referred to as analytic and holistic/synthetic listeners (von Helmholtz, 1885; Smoorenburg, 1970; Houtsma, 1979), henceforth referred to as *spectral* and *fundamental* listeners, respectively. Spectral listeners primarily focus on the individual harmonic constituents, they “decompose the sound” (Schneider and Wengenroth, 2009, p. 316), while fundamental listeners are attuned to the relationship between all the higher harmonics present in the signal, which supports their perception of the fundamental frequency (Rousseau et al., 1996; Laguitton et al., 1998; Seither-Preisler et al., 2007). According to von Helmholtz, (1885), for fundamental listeners, it is as if the harmonics “fuse into the whole mass of musical sound” (Schneider and Wengenroth, 2009), hence his choice of the term “holistic” or “synthetic” to refer to this type of listening mode. While in practice, few listeners perform uniquely at the absolutes of one or the other type (Ladd et al., 2013), the perceptual bias may lead to different interpretations of perceived pitch values in particular contexts. On the one hand, the perception of the fundamental frequency is supported by so-called combination tones generated in the cochlea (Plomp, 1976). These tones differ across individual listeners (Probst et al., 1986). On the other hand, results of structural MRI studies suggest that the bias is, at least partly, due to a right-/leftward asymmetry of gray matter volume in the lateral Heschl's gyrus (Schneider et al., 2005a,b; Wong et al., 2008), the so called “pitch processing center” (Griffiths, 2003). In particular, larger volumes of right Heschl's gyrus seem to be associated with spectral perceptual bias, while the left Heschl's gyrus has been linked to changes in the *F*_0_ modulation and temporal information (Schneider et al., 2005a,b; Warrier et al., 2009). Until fairly recently, the perceptual bias has mainly been examined in the context of musical psychoacoustics. The research outcomes of (Wong et al., 2008), however, may be interpreted as support for the claim that it may also affect linguistic performance. In their study of lexical tone perception, listeners who performed worse in a word identification task involving vowels with superimposed tones showed a smaller Heschl's gyrus volume on the left than listeners who performed better. Given the tight link between perception and production, recently implemented in the “forward-model” of (Pickering and Garrod, 2013) where internal simulation of input utterances facilitates comprehension and shapes phonetic output, we assume that advantages in the perception of *F*_0_ might improve its imitation. In other words, fundamental listeners may have a better capacity to adapt their pitch to their communication partners than spectral listeners.

**Figure 1:**
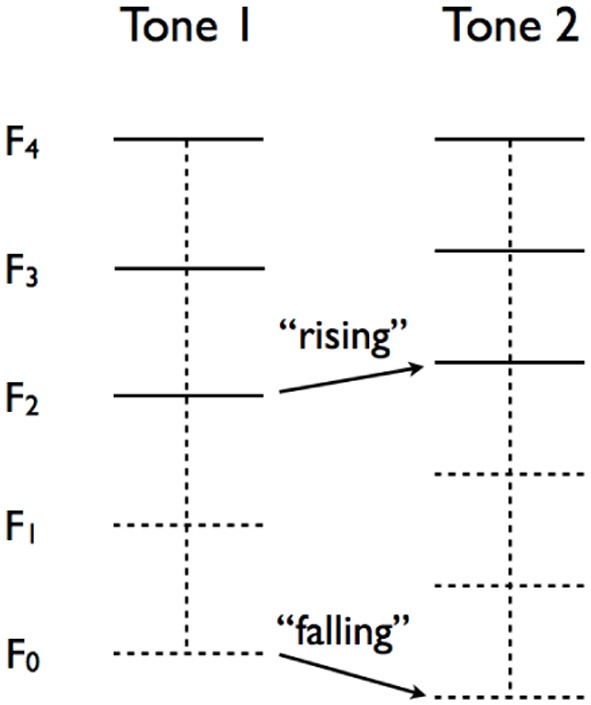
**Illustration of an ambiguous two-tone sequence to determine auditory perception bias**. The sequence has a falling (missing) fundamental *F*_0_ and a rising lowest partial *F*_*lp*_ The horizontal lines represents the partials of the tone. The higher partials are physically present (solid lines) and the lower partials are missing (dashed lines).

In what follows, we present the results of a production study conducted to determine the effect of auditory perception bias on automatic pitch imitation in a classical shadowing task. Listeners' perception bias was determined with the help of missing fundamental stimuli, an idea that originated with (Smoorenburg, 1970) who introduced a forced-choice task involving sequences of two complex tones. In the task, participants are presented with a sequence and asked to indicate if the perceived pitch is rising or falling. The crux of the task is that the tone sequence is designed to have an ambiguous pitch change. Each complex tone is created from *m* partials *F*_*n*_, *F*_*n* + 1_, … *F*_*n* + *m* − 1_, (*n* is an integer, *n* > 0), without the fundamental *F*_0_. The ambiguity arises from the opposite changes of the (missing) fundamentals (*F*_0_) and the (physically present) lowest partials (*F*_*lp*_). When the subsequent fundamentals *F*_0_ are rising, the lowest partials *F*_*lp*_ are falling, and vice versa. Representing the partials of the first and second tones by *F*^1^ and *F*^2^, respectively, fundamental listeners will perceive the change in pitch Δ*P*_*f*_ by computing Δ*P*_*f*_ = (*F*^2^_*k* + 1_ − *F*^2^_*k*_) − (*F*^1^_*k* + 1_ − *F*^1^_*k*_) (*k* ϵ {*n*, *n* + 1, … *n* + *m* − 2}) in order to estimate *F*^2^_0_ − *F*^1^_0_. Spectral listeners will rely on Δ*P*_*sp*_ = *F*^2^_*lp*_ − *F*^1^_*lp*_ to determine if the pitch is rising or falling. Figure 1 illustrates an ambiguous tone sequence. The sequence depicted has a falling *F*_0_ (Δ*P*_*f*_ < 0) and a rising *F*_*lp*_ (Δ*P*_*sp*_ > 0).

Since the early work of Smoorenburg, (1970), the listener type task has been frequently employed to study how acoustic variables (e.g., *F*_0_-value, ΔP-value, number of partials) affect the perception of pitch (Plomp, 1965; Ladd et al., 2013). Schneider et al., (2005b) and (Seither-Preisler et al., 2007) used the task to explore the distribution of listener types in relation to musical training. In both studies, participants were presented with a number of ambiguous-sequence stimuli. The proportion of stimuli to which a fundamental or spectral pitch change was perceived by the participants, defined the so-called Coefficient of Sound Perception Preference' (δ_*p*_), a value ranging from −1 (all stimuli perceived as spectral) to +1 (all stimuli perceived as fundamental). To prevent the emergence of combination tones that arise at the level of the cochlea (Terhard, 1974), Schneider et al., (2005b) presented tones at low intensity and Seither-Preisler et al., (2007) added masking noise to their stimulus sequences. Given that the perception thresholds of combination tones vary with the individual (Plomp, 1976), following (Ladd et al., 2013), we made use of stimuli without masking in an attempt to include possible effects of cochlear mechanisms on the perception and production of pitch in speech.

## Materials and methods

### Participants

Eighty-eight Dutch native speakers (67 females) between the age of 17–25 years (*M* = 20.48, *SD* = 2.12) participated in the experiment for course credit. None of them reported any hearing difficulties. Fourteen of the participants were left-handed; about one half of the experimental group described their musical proficiency as low to average, the other half assessed their proficiency as high to professional. Male and female were divided equally between the two experimental conditions. Prior to the experiment which had received an approval from the ethical committee, participants provided their written informed consent.

### Measuring auditory perception bias

Participants' auditory perception bias was determined with a variation of the psychoacoustic perceptual test described in Smoorenburg, (1970), Laguitton et al., (1998), Schneider et al., (2005b), and Seither-Preisler et al., (2007). For the perceptual test, we constructed 36 pairs of complex harmonic tones, all 160 ms long, that consisted of 2–4 harmonics, with the same harmonic composition as employed by Laguitton et al., (1998). Participants were asked to categorize 18 perceptually ambiguous stimuli sequences consisting of two complex tones, tone 1 and tone 2 as illustrated in Figure 1. All tones were composed of a number of upper harmonic tones with the same highest harmonic but different levels of virtual fundamental pitch (derived from the harmonics as the best fit) and spectral pitch (based on the lowest harmonic). The other 18 stimuli served as control trials in that their interpretation was unambiguous but helped to determine a participant's level of attention to the task. Listeners were instructed to categorize each experimental stimulus (tone pair) as either “rising” or “falling,” depending on their perception of the sequence. Based on their answers, we calculated their individual “Coefficient of Sound Perception Preference” (δ_*p*_) using the equation δ_*p*_ = (*F* − *Sp*)/(*F* + *Sp*), where F is the number of virtual fundamental classifications and Sp the number of spectral classifications. We calculated the “Listener Attention Coefficient” (δ_*A*_) as the proportion of correctly categorized unambiguous stimuli. In order to test the validity of the perceptual test, we repeated the measurement approximately 1 month later under the same conditions with a subset of the participant set (*N* = 64). In the analyzes presented below, we report the overall results for all experimental stimuli (δ_*p*_), as well as the results for stimuli where the lowest present component frequency *F*_*n*_ > 1000 Hz, δ_*p*1000_. The 1000 Hz value is arguably the highest frequency at which *F*_0_ could be produced by a human voice and also the approximate maximal value at which the missing fundamental phenomenon occurs (Fletcher, 1924). Stimuli with *F*_*n*_ > 1000 Hz thus arguably support the perception of the missing fundamental.

### Speech imitation task

The shadowing task took place immediately after the psychoacoustic task. It consisted out of eight declarative and eight interrogative sentences uttered by four different model talkers (two male, two female) in a between-subject design in order to maximize exposure to the model speaker's voice and thus increase chances of possible imitation. The 16 sentences were recorded four times: in the first and fourth block, the participants read the sentences in a randomized order (same for all participants) from a PowerPoint slide; the declarative and interrogative sentences were presented in a mixed design. In the second and third block, they were asked to repeat the sentences as they were presented to them (in auditory modus only), through high quality headphones (Sennheiser HMD26-600-7). The participants were not explicitly instructed to imitate the speakers' pronunciation but simply to repeat the utterances. They were randomly assigned to one of two between-subject conditions (filtered vs. unfiltered). In the filtered condition, participants heard recordings that were filtered with an order nine high-pass Butterworth filter with cutoff frequency of 300 Hz, using Matlab's Signal Processing Toolbox. The participants in the unfiltered condition heard full speech recordings.

### Automatic pitch estimation

An initial set of analyzes was performed on the whole corpus with a subsequent more detailed analysis of a shorter speech segment. The recordings were segmented per utterance and analyzed using the autocorrelation method, see, e.g., Rabiner et al., (1976), implemented in Matlab using a frame length of 10 ms with 5 ms overlap, and a frequency range of 50–500 Hz. For the whole corpus, we computed five statistical descriptors of *F*_0_: the mean value, the maximum, the minimum, the range (max–min) and the standard deviation. The degree of *F*_0_ imitation was determined by assessing the *z*-score of the absolute difference between the model speaker's *F*_0_ descriptor and the participant's *F*_0_ descriptor in the first block (*D*_1_, baseline) and the second and third block (first and second shadowing, *D*_2_ and *D*_3_, respectively). We defined two measures of imitation, *F*_0_ Imitation_1_ = *D*_1_ − *D*_2_ and *F*_0_ Imitation_2_ = *D*_1_ − *D*_3_. The statistical analyses were conducted with the IBM SPSS Statistics software v.2.0.

## Results

In this section we present the results of our experiment in four parts. First, we present the descriptive values of the “Coefficient of Sound Perception Preference” δ_*p*_ in the first and second measurement. Second, all results obtained in the first measurement are compared to global—sentence level—imitative behavior. Third, using smaller speech segments, a correlation analysis is performed on the psychoacoustic and socio-demographic variables to determine the inclusion of variables in a regression analysis. Finally, the results of a hierarchical multiple regression analysis are presented that relate auditory perception bias to *F*_0_ imitation.

### Coefficient of sound perception preference

**Figure 2:**
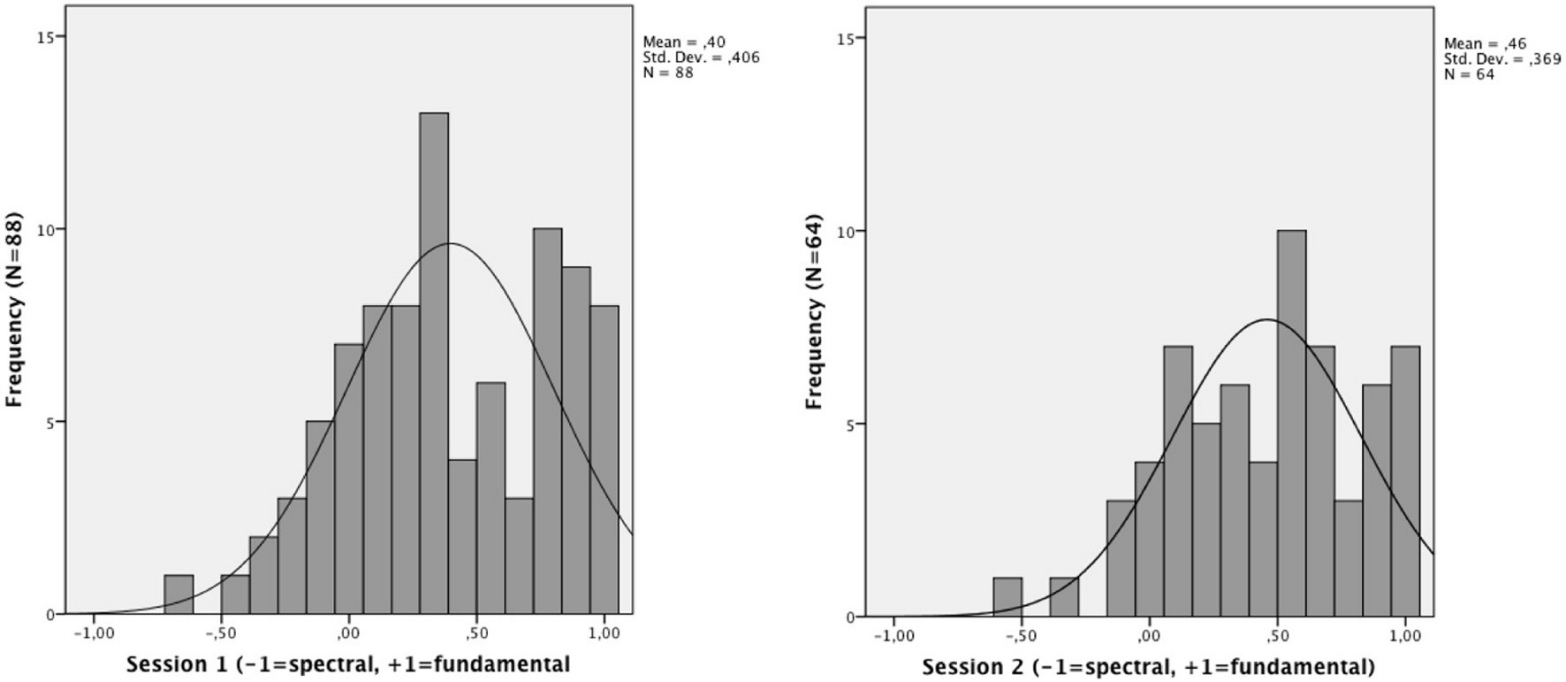
**Distribution of δ_*p*_ during the first session and second session of the psychoacoustic task**.

The Shapiro–Wilks test of normality revealed that the coefficient δ_*p*_ was not normally distributed: the majority of the participants performed as fundamental listeners (Mean δ_*p*_ = 0.397, *SD* = 0.406). For a distribution of the δ_*p*_, see Figure 2. A comparison of the first and the second measurement showed that repeated exposure to the ambiguous stimuli resulted in a shift toward the fundamental bias, with a significant correlation between the two measurements (Spearman's ρ = 0.69, *p* < 0.001). The test-retest correlation was comparable to that provided by Ladd et al., (2013). The difference between the two measurements was marginally significant with Wilcoxon Signed Ranks test, *Z* = − 1.87, *p* = 0.06. In order to explore the possibility that the difference between the first and the second measurement of participants' perception was due to the level of attention devoted to the task, we compared the absolute difference between the first and the second δ_*p*_, δ_*p*_1, and δ_*p*_2, to the attention coefficient δ_*A*_. The correlation between the attention coefficient δ_*A*_ in the first measurement and the | δ_*p*_1 − δ_*p*_2 | was significant (Spearman's ρ = −0.35, *p* < 0.01), indicating that poor attention to the task during the first measurement may have been the reason for the observed shift in δ_*p*_ (given that the shift was in the direction from “undecided” to a more “pure” type of perception, see Figure 2). As pointed out by (Seither-Preisler et al., 2007), however, who reported a similar result attributed to repeated exposure, an effect due to learning cannot be excluded (no measures of δ_*A*_ were provided in their study). In the subsequent analysis relating speakers' perceptual bias to their capacity to imitate *F*_0_, we used the value of δ_*p*_ collected during the first measurement, i.e., in the same session as the shadowing task.

### Sentence-level imitation

In the initial global analysis of the whole corpus, for each pitch value, we conducted two statistical tests, one with the full participant sample and one where we only included those participants who had more than 90% correct (less than 2 mistakes of the total of 18 trials) in the categorization of the unambiguous stimuli in the psychoacoustic task (*N* = 41 in total, with *N*_full signal_ = 22 and *N*_filtered_ = 19) and were thus assumed to be reliable as listeners. Tables 1, 2 give an overview of the correlations between *F*_0_ imitation (rows expressed in Hz) for the five descriptors (columns) and the Coefficient of Sound Perception Preference, δ_*p*_, split by condition (with full signal, viz. Table 1, and with the signal under 300 Hz filtered out, viz. Table 2).

**Table 1:** **Pearson product-moment correlations between δ_*p*_ and *F*_0_ imitation in the full signal condition (first value for all participants, second value for participants with *PC*_δ*A*_ > 90)**.

**Variable**	**Mean *F*_0_**	***F*_0_ Max**	***F*_0_ Min**	***F*_0_ Range**	***F*_0_*SD***
*F*_0_ Imitation_1_	−0.06/−0.17	−0.08/0.043	−0.23^‡^/−0.15	0.30^*^/0.37^*^	0.20^‡^/0.41^*^
*F*_0_ Imitation_2_	−0.01/−0.16	0.11/0.16	−0.18/−0.07	0.23^‡^/0.33	0.14/0.39^*^

PC = “% correct.”

‡ *p < 0.10*,

* p < 0.05.

**Table 2:** **Pearson product-moment correlations between δ_*p*_ and *F*_0_ imitation in the filtered condition (first value for all participants, second value for participants with *PC*_δ*A*_ > 90)**.

**Variable**	**Mean** ***F*_0_**	***F*_0_ Max**	***F*_0_ Min**	***F*_0_ Range**	***F*_0_,*SD***
*F*_0_ Imitation_1_	0.03/0.37	−0.07/0.44^*^	−0.03/0.06	0.17/0.33^*^	0.22^‡^/0.33
*F*_0_ Imitation_2_	−0.02/0.43^*^	−0.23^‡^/0.42^*^	−0.27^*^/−0.07	−0.09/0.40^*^	−0.04/0.34

PC = “% correct.”

‡ *p < 0.10*,

* p < 0.05.

The results of the global analyses suggested that, overwhelmingly, participants who performed reliably on the non-ambiguous task and scored higher in the direction of fundamental listeners imitated the model speakers' pitch to a higher degree, especially in the condition with filtered speech signal. Given that the analyses were performed on full utterances, however, they might have been less likely to capture *F*_0_ imitation that typically occurs on individual segments (especially, vowels) and less reliable given that local minima and maxima (that, in turn, affect the range and SD) may be outliers in the signal without communicative significance. Therefore, we proceeded with a more fine-grained analysis of a subset of the corpus, in which we also included socio-demographic variables collected in the experiment.

### Vowel segment imitation

In order to limit the size of the corpus collected in the shadowing task, we randomly selected one of the interrogative sentences for the subsequent analyses, focusing on its initial voiced segment. The choice of an interrogative sentence was driven by the assumption that (1) imitation is likely to occur at sentence-initial boundaries immediately following the model talker's output (Nilsenova and Nolting, 2010), and (2) polar (yes/no-) interrogatives that are context-free (no particular word in the interrogative is in focus) are intonationally marked by a pitch excursion (van Heuven and Haan, 2002), i.e., in this case, on the finite verb that is sentence-initial due to subject-verb inversion. An automatic analysis of pitch was performed on the initial occurrence of the vowel /a/ in the sentence. The segment fundamental frequency was determined by averaging over the *F*_0_ values of approximately the first half of the initial vowel in order to avoid right vowel boundary detection errors.

Preliminary data analysis was conducted to identify potential covariates, using both demographic and psychoacoustic variables. Chi-square tests indicated that there were no significant differences between the full speech and high-pass filtered condition with respect to participant gender and handedness, there was also no significant difference between stimulus voice (two male, two female) and participant gender. Non-parametric Mann–Whitney Tests for variables without normal distribution indicated no significant difference between the experimental conditions with respect to musicality [determined on the basis of a self-reported evaluation on an 11-point scale, anchored at 0 (no experience) and 10 (professional musician)], age, δ_*p*_ (sound perception preference), δ_*A*_ (listener attention) and δ_*p*1000_ (sound perception preference for stimuli above 1000 Hz). A zero-order correlation analysis assessed the relationship between demographic and psychoacoustic variables. The purpose of the matrix was to determine which variables might affect degrees of imitation and could thus be included in the regression analysis. As seen in Table 3, there was a significant correlation between musicality and δ_*p*_ (*r* = 0.51, *p* < 0.001), δ_*A*_ (*r* = 0.49, *p* < 0.001) and δ_*p*1000_ (*r* = 0.46, *p* < 0.001); participants with more musical experience performed with a more fundamental perceptual bias with respect to stimuli with a missing fundamental and scored higher on categorizing non-ambiguous acoustic stimuli as well. There was also a significant correlation between δ_*p*_ and δ_*A*_ (*r* = 0.47, *p* < 0.001) and δ_*p*1000_ and δ_*A*_ (*r* = 0.39, *p* < 0.001), more fundamental perceptual bias was related to a better performance on the non-ambiguous stimuli. The two ways of assessing auditory perception bias, δ_*p*_ and δ_*p*1000_, were significantly correlated (*r* = 0.94, *p* < 0.001). A trend for significance was observed in the relation between the first *F*_0_ imitation and the experimental condition and between gender and the second *F*_0_ imitation (significant with α < 0.10). In addition to the correlation tests, we also explored the effect of the categorical variables (Condition, Gender and Handedness) on the measures of the Listener Attention Coefficient, the Coefficient of Sound Perception Preference, the Coefficient of Sound Perception Preference above 1000 H, *F*_0_ Imitation_1_ (first shadowing block) and *F*_0_ Imitation_2_ (second shadowing block). Gender and handedness had no effect on any of the measures. There was a marginally significant effect of condition on *F*_0_ Imitation_1_ (*t*_(86)_ = −1.81, *p* = 0.07) with a lower degree of imitation in the filtered condition compared to the full speech condition. There were no other significant effects of condition. Based on the results of the correlation analyses which suggested a stronger link between δ_*p*1000_ and imitation, only δ_*p*1000_ was included as a covariate in the primary statistical modeling of the first *F*_0_ imitation (first shadowing, i.e., second block in the session) in the two experimental conditions.

**Table 3:** **Zero-order Pearson product-moment correlations among psychoacoustic variables and the socio-demographic variables**.

	**Variable**	**1**	**2**	**3**	**4**	**5**	**6**	**7**	**8**	**9**	**10**
1.	Condition	–									
2.	Age	−0.02	–								
3.	Gender	0.08	−0.14	–							
4.	Handedness	−0.06	0.01	0.12	–						
5.	Musicality	−0.07	0.18	0.07	−0.18	–					
6.	δ_*A*_	0.06	0.07	−0.07	−0.04	0.49^**^	–				
7.	δ_*p*_	−0.09	0.11	0.04	0.09	0.51^**^	0.47^**^	–			
8.	δ_*p*1000_	−0.10	0.06	0.01	0.04	0.46^**^	0.39^**^	0.94^**^	–		
9.	*F*_0_ Imitation_1_	0.19^‡^	−0.03	0.11	0.12	−0.03	0.05	0.17	0.24^*^	–	
10.	*F*_0_ Imitation_2_	0.15	−0.05	0.18^‡^	0.10	0.00	0.06	0.17	0.18	0.62^**^	–

“Condition” was dummy-coded to compare the effect of frequency filtering with other responses (1 = filtered, 0 = full speech). “Gender” was dummy-coded to compare the performance of male and female listeners (1 = female, 0 = male). “Handedness” was dummy-coded to compare the performance of left- and right-handers (1 = right, 0 = left). δ_A_, Listener Attention Coefficient; δ^p^, Coefficient of Sound Perception Preference; δ_p1000_, Coefficient of Sound Perception Preference above 1000 Hz.

‡ *p < 0.10*,

* *p < 0.05*,

** p < 0.001.

**Figure 3:**
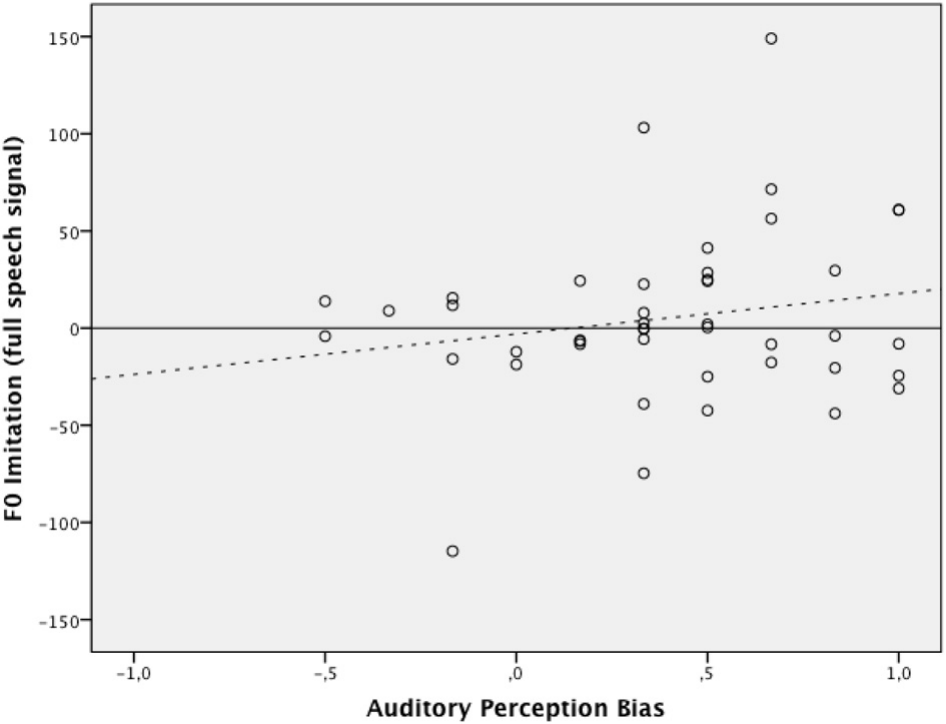
**The relation between auditory perception bias above 1000 Hz and the degree of *F*_0_ imitation in the condition with full speech signal (dotted line indicating trend)**. The x-axis represents the auditory perception bias expressed as δ_*p*_(1000). The y-axis expresses the difference between *D*_1_, the absolute difference between the model speaker's *F*_0_ and the participant's *F*_0_ in the first (baseline) block, and *D*_2_, the absolute difference between the model speaker's *F*_0_ and the participant's *F*_0_ in the second (first shadowing) block; a positive value here indicates imitation and a negative value indicate divergence.

**Figure 4:**
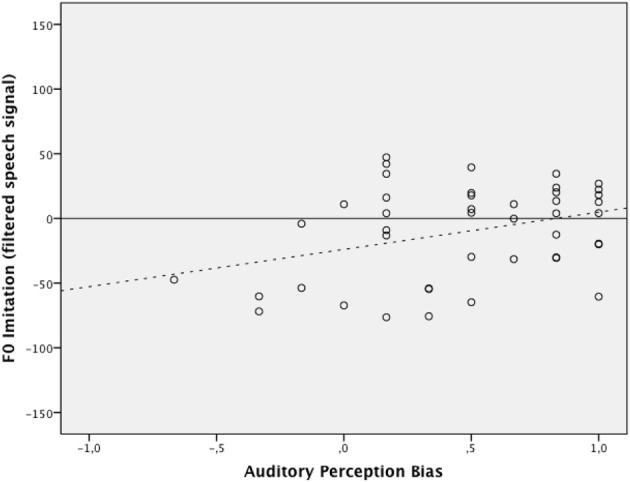
**The relation between auditory perception bias above 1000 Hz and the degree of *F*_0_ imitation in the condition with filtered signal (dotted line indicating trend)**. The *x*- and *y*-axis represent the same measures as in Figure 3.

Hierarchical multiple regression was used to establish the incremental value of auditory perception bias when predicting the level of *F*_0_ imitation in a condition with high-pass band filtered speech and in a condition with full speech signal. The regression model consisted of two blocks and assessed the additional variance explained with the estimation of each added block. At Block 1, the centered values of δ_*p*1000_ and experimental condition were entered simultaneously. This block resulted in a significant overall model, *F*_(2, 85)_ = 4.87, *p* = 0.01, accounting for 10% of the variance in the imitation scores. The interaction effect between δ_*p*1000_ and experimental condition was created by multiplying the mean-centered values of each individual variable and then was entered at Block 2 along with all variables entered at Block 1. Results again indicated an overall effect for the model, *F*_(3, 84)_ = 3.27, *p* = 0.03, explaining an additional variance of 0.2%. The δ_*p*1000_ by experimental condition interaction term did not significantly predict the imitation scores after controlling for covariates and main effects (*b* = −8.02, *p* = 0.69). Figures 3, 4 graphically display the main effects of δ_*p*1000_ and condition on *F*_0_ imitation. The y-axes express the difference between *D*_1_, the absolute difference between the model speaker's *F*_0_ and the participant's *F*_0_ in the first (baseline) block, and *D*_2_, the absolute difference between the model speaker's *F*_0_ and the participant's *F*_0_ in the second (first shadowing) block; a positive value here indicates imitation and a negative value indicate divergence. The figures show that more fundamental listeners were better at imitating the fundamental frequency in the model speaker's voice. Fully tabulated results of the hierarchical regression model are presented in Table 4.

**Table 4:** **Results of the hierarchical regression model**.

**Variable**		***b***	***SE***	β	**Adjusted** ***R*^2^**	Δ***R*^2^**
Step 1					0.08	0.10^**^
	δ_*p*1000_	26.07	10.02	0.26^**^		
	Filter condition	17.46	8.33	0.22^*^		
Step 2					0.07	0.00^*^
	δ_*p*1000_	24.79	10.10	0.26^*^		
	Filter condition	17.44	8.37	0.22^*^		
	δ_*p*1000_ by filter condition	−8.02	20.19	−0.04		

δ_p1000_, coefficient of sound perception preference above 1000 Hz.

* *p ≤ 0.05*,

** p ≤ 0.01.

## Discussion

Our findings suggest that auditory perception bias can partly account for the individual variation found in earlier pitch imitation studies. In a shadowing task, fundamental listeners showed a better capacity to imitate the vocal pitch of the model talkers, especially in a condition where the region between 0–300 Hz has been filtered out and information about *F*_0_ had to be derived from the higher frequencies (akin to telephone speech). These results can be used in future studies on speech imitation abilities, e.g., to explore phenomena such as phonetic (pronunciation) talent (Lewandowski, 2009).

Our findings of individual differences in listener's sensitivity to tone sequences may be related to those of Semal and Demany, (2006), who found some listeners to be able to detect changes in tone sequences, while unable to indicate the direction of change (upward or downward). Future studies should address the relation between individual differences in sensitivity to pitch direction and in auditory perception bias. At this point it is unclear what is causing the individual differences in auditory perception bias. As stated in the Introduction, Schneider et al., (2005b) found neuroanatomical differences in the lateral Heschl's gyrus to be associated with perception bias. However, the differences may very well be of a more peripheral origin, i.e., reflecting individual differences in cochlear responses. In particular, non-linear interactions in the cochlea may give rise to so-called combination tones (Plomp, 1965). When stimulated with a tone consisting of the *n*-th and (*n* + 1)th harmonic, the cochlea may generate tones at a frequency corresponding to that of the missing fundamental. It is important to stress that the generated tone is physically present because it is generated in the cochlea, rather than being extracted from the harmonics (as is the case for the missing fundamental). Plomp, (1965) claimed that combination tones are inaudible for “usual levels” of speech and music and that the same applies to the perception of the missing fundamental. Notwithstanding this claim, in his study of individual differences in (what we call) auditory perception bias, Smoorenburg, (1970) effectively suppressed the perception of combination tones by superimposing masking noise bands centered at the combination-tone frequencies. Apparently, Smoorenburg, (1970) was concerned about a potential interfering effect of combination tones in the determination of listener type. Given that in the experiment reported here, the stimuli were presented without masking noise, the participants may have perceived physically generated tones at the level of the missing fundamental. The generation of combination tones could have lead to overestimates of δ_*p*_, because spectral listeners may perceive the combination tone instead of a reconstructed fundamental (as fundamental listeners do), thus explaining the skewed distribution in both first and second measurement of the perception bias. On the one hand, the presence of combination tones may invalidate the determination of listener type. On the other hand, combination tones are an inevitable byproduct of naturally occurring sounds. Cochlear dynamics generate combination tones which affect further cortical processing and anatomical correlates (i.e., lateral Heschl's gyrus). As such, the auditory perception bias as measured in our experiment takes into account individual variations in sensitivity to combination tones. In general, the potential role of combination tones in the definition and study of listener types deserves further attention. Ladd et al., (2013) pointed at the methodological differences in earlier studies of listener type performed by Schneider et al., (2005b) and Seither-Preisler et al., (2007), but did not identify the use of masking noise (or other means to suppress combination tones) as a main methodological difference between their study and both earlier ones. In our future work, we aim at a detailed investigation of the role of combination tones in auditory perception bias.

### Conflict of interest statement

The authors declare that the research was conducted in the absence of any commercial or financial relationships that could be construed as a potential conflict of interest.
